# Amid COVID-19: the importance of developing an positive adverse drug reaction (ADR) and medical device incident (MDI) reporting culture for Global Health and public safety

**DOI:** 10.1186/s40545-020-00219-1

**Published:** 2020-05-18

**Authors:** Ali Elbeddini, Aniko Yeats, Stephanie Lee

**Affiliations:** 1 Winchester District Memorial Hospital (WDMH), 556 Louise St, Winchester, ON K0C2K0 Canada; 2 Leslie Dan Faculty of Pharmacy, University of Toronto, 144 College Street, Toronto, Ontario M5S 3M2 Canada

**Keywords:** COVID-19, Reporting, Adverse drug reaction, Patient safety, Medical device incident

## Abstract

Amid **COVID**-**19** Crisis, reporting adverse drug reactions (ADRs) and medical device incidents (MDIs) to Health Canada or health authorities in every country is crucial for monitoring medication safety and improving public health. Health Canada, for example, through their online database, has facilitated the process of reporting side effects relating to drugs and medical devices. However, several patients and health care professionals still fail to voluntarily report adverse events. For health care providers, some barriers to reporting may include fear of negative feedback, apathy, legal concerns, and uncertainty about whether an incident qualifies as an ADR.

In the current **COVID-19** Crisis, it is especially important for health care providers to be diligent about reporting Adverse Drug Reactions (ADRs), since misinformation propagated by the media is causing patients to misuse certain medications. We need to shift the current thought process about ADR reporting in order to encourage a positive reporting culture by patients and health care providers.

## Introduction

Is under-reporting is a concern?

In Canada, we are underreporting ADRs, with less than 10% of ADRs being reported [1–3]. Under-reporting is a global problem [4]. In lower and middle-income countries, a priority is securing access to drugs for treatment and limited resources are available for pharmacovigilance [5].

There are a number of considerations related to ADR reporting. For instance, health care providers are more likely to report ADRs that are serious and unexpected. It has also been observed that new drug products tend to generate more reports than older products [6].

As observed in a study by Li Q et al., healthcare professionals in Wuhan, China lack the understanding of properly reporting ADRs [6]. The current **COVID**-19 Crisis has brought about fear and uncertainty in many, resulting in an increased demand for effective antiviral therapies against **COVID-**19. There are several potential therapies coming out against **COVID**-19, however patients and healthcare professionals must understand that these are only preliminary trial results and they need more study to fully understand their efficacy and side effect profiles [10]. Unfortunately, the media and certain public figures promote therapies that are not yet proven leading to people taking medications inappropriately and experiencing some severe ADRs. In an era where medication misinformation is rampant, quality pharmacovigilance has become more important than ever.

Healthcare professionals generally understand the value of reporting; however, many are not fully aware of the mechanisms for reporting [6, 7]. Some of the main reasons for not reporting include: lack of time, complex documentation, lengthy reporting procedures, failure to recognize an ADR, patient confidentiality concerns, and fear of blame. There is also a lack of routine, structured reporting and shared motivation [7].

### Opportunities

There has been a debate amongst healthcare providers and the public regarding what, when and how to report ADRs. There is an opportunity to promote ADR reporting and learning through increased educational sessions at schools, universities, medical associations, and in community outreach programs.

There is also a need for more guidance from the professional regulatory authorities and associations. Effective reporting and education related to ADRs is a component of public health and safety. The sample size of the patient population and the limited duration of clinical studies do not provide the full evidence of a drug’s safety profile. Post-market surveillance is critical. When a larger population of patients use a medication, rare ADRs can be captured if they get reported.

**Fig. 1:**
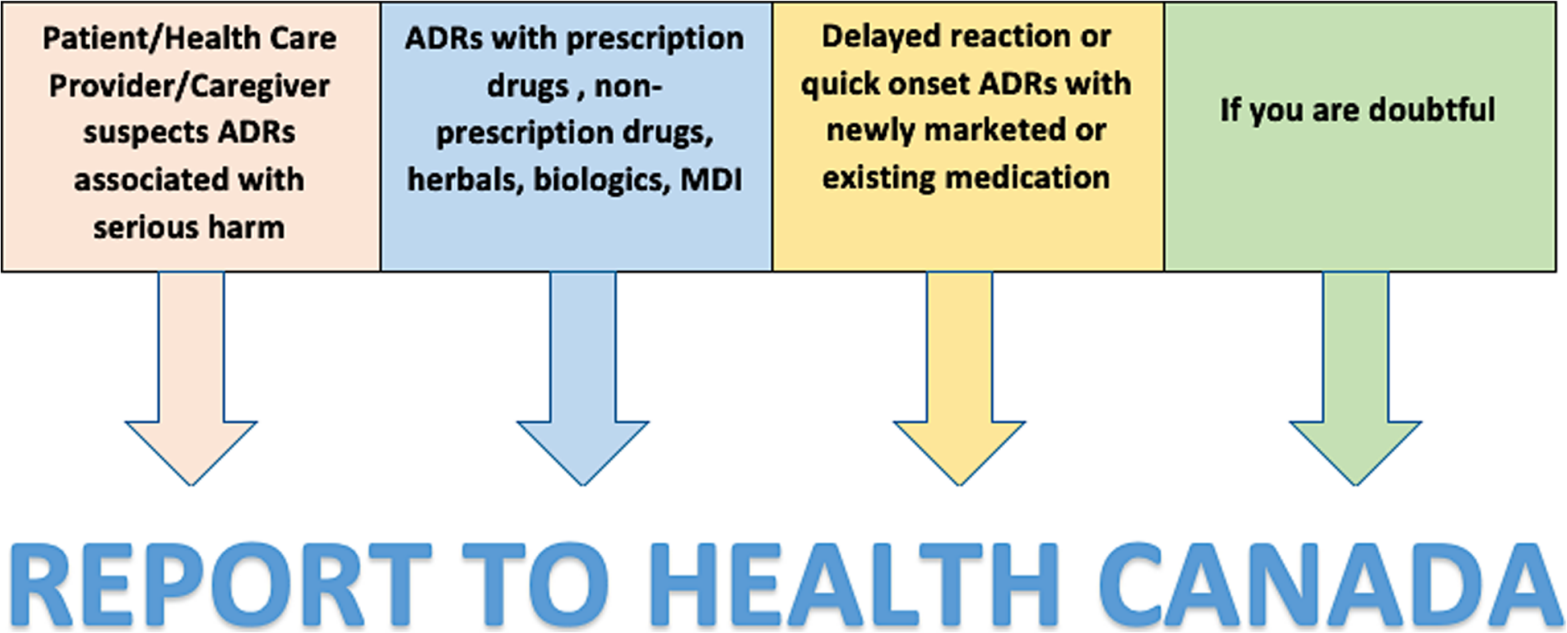
Examples of Instances for ADR reporting. Several types of ADRs warrant reporting to Health Canada. All suspected serious ADRs must be reported, as well as delayed or quick onset ADRs. Even when an ADR is not certain, it is best practice to report the event to Health Canada

Figure 1 newly marketed medications and devices are important to monitor closely for safety. The side effects of a medication could be well known, but there might be uncertainty about the serious reactions that warrant reporting. Serious reactions requiring reporting are those that are fatal, life-threatening, disabling, or require hospitalization. It is always important to keep an open channel of communication between patients, physicians and pharmacists in order to ensure that patients are clear about the reporting process. Delayed reactions must also be monitored, as these reactions may not occur immediately after the administration of a medication. Delayed reactions may take several days to weeks to appear. ADRs in children and the elderly are especially important to report since they are more susceptible to the adverse effects of drugs. Elderly populations tend to take multiple medications for a wide range of comorbidities, and they may also have altered pharmacokinetic and pharmacodynamic responses to medications. Patients are most affected by medications: therefore it is important to collect their experiences and analyze them. At each communication with a patient, whether in the hospital or community setting, we need to incorporate medication safety questions into our discussions [11]. ADRs are a major concern for patients when they start new medications.

### Adapting to Health Canada’s new mandate will require important changes to ensure quality reporting

Unfortunately, healthcare settings are plagued with heavy workloads, multitasking, constant interruptions, and labor shortages [8]. For this reason, patients might feel that they are a burden and may hesitate when it comes to reporting any side effects to medications. Patients must be made aware of the “5 Questions to Ask about Your Medications” [11]. Having these questions on a poster board in the pharmacy or hospital might help to remind and encourage patients to actively seek answers to any questions they may have about their medications. If patients are not comfortable speaking to their own health care provider about their medication concerns, there should be a staff member available in the hospital that could readily document their medication concerns and relay that information to a qualified health care professional. Improving the safety of medications is one of the most significant ways in which we can enhance the quality of healthcare. ISMP Canada is an organization put in place to advance medication safety and to ensure reliable medication practices in all points of healthcare. To further improve ADR reporting, ISMP Canada has made an anonymous reporting tool available to health professionals and patients, where they could report any medication incidents [9].

The new regulation mandating the reporting of serious ADRs and MDIs by hospitals will have a large administrative and operational burden on hospital pharmacists and other hospital workers. Furthermore, mandatory reporting may adversely impact the hospital budget as well as the quality of care offered to patients [8]. Therefore, the involvement of a senior leadership team dedicated to complying with the new regulations will greatly facilitate quality reporting. It would be detrimental to patient care if the current front-line hospital providers were to be diverted from their current roles in order to invest more time into reporting ADRs to Health Canada.

Hospitals are required to report serious ADRs and MDIs for prescription and non- prescription medications, biologic drugs, radiopharmaceuticals, disinfectants, medical devices, and drugs needs for emergency public health purposes [1]. Senior leadership teams could be trained on using the various reporting platforms quickly and efficiently so that this new mandate does not compromise the workflow at the hospital. The front-line healthcare professionals must document any serious ADRs as always, however the designated leadership team could be given specific internal roles and responsibilities at the hospital to evaluate these ADRs, categorize them, and determine whether they warrant reporting to Health Canada. It is important to note that hospitals are currently required to report all documented serious ADRs and MDIs, regardless of whether the event initially occurred in the hospital [1]. These reports must be as thorough and as complete as possible in order to help Health Canada’s assessment of causality. Furthermore, if new information becomes available relating to a patient’s previous ADR or MDI, the hospital must send follow-up information to Health Canada [1].

Moreover, leadership teams could be involved in implementing a tracking system for the reports made in the hospital. This would be to ensure facilitated communication between Health Canada and the hospital, in case there are any follow-up questions pertaining to an ADR report.

Furthermore, one of the key roles of pharmacists in the hospital setting is to conduct Best Possible Medication Histories (BPMHs), which means that they review the medications that the patient takes prior to coming to the hospital. During this standardized interview, pharmacists can pick up on any drug therapy problems, including adverse effects to medications. As such, having pharmacists in the leadership team conducting BPMHs could be an efficient way in interviewing patients for potential ADRs or MDIs. This would ensure that there is a standardized protocol in place to gather all of this pertinent information in an efficient manner.

Some future steps could be to conduct surveys in all provinces to see how this new regulation has been implemented so far. We can also analyze the mechanisms that the various provinces use to report, and subsequently compare the efficiency and quality of individual province reports.

### Summary

With the newly implemented regulations for mandatory reporting of serious ADRs, there is an opportunity to utilize technologies and systems to facilitate reporting and capture big data.

Health care providers have many demands on their time and this is an important factor that warrants discussion at all levels in the organization/facility.

This work requires integration of information in technology systems (e.g. electronic health record, incident reporting systems and or health record coding processes) to enable different systems to submit information to Health Canada. Health Canada is providing data transfer mechanisms for submission of data [1].

Organizing an ADR awareness week and including a national education day throughout Canada could help to engage and educate more Canadians. Creating a positive reporting culture is a shared responsibility and requires more than one individual.

Healthcare providers may have a different opinions when it comes to deciding whether certain cases require submission as a serious ADR. An email back to the identifier or reporter will work as a guidance and education tool for the next reporting, since the one reporting will know whether or not Health Canada classified their event as an ADR. This feedback function will make health care providers feel appreciated and heard, which will allow for an open channel of communication between reporters and Heath Canada. Creating a culture of reporting and culture of safety is important to encourage all to report with no fear.

The communication and collaboration between health care authorities and identifiers/reporters will be instrumental in increasing the rate of reporting. The pharmacist has a duty to report ADRs, but so do patients. Medical and pharmacy schools also have a responsibility for teaching about ADR reporting and implementing a culture of public safety to educate students. Distributing posters at health care facilities will be an important first step in opening a discussion about ADR reporting.

Additionally, each hospital needs to work out all logistics that become a barrier to reporting by creating free time to report and receive proper education. There seems to be a misconception that reporting an ADR is equivalent to reporting the side effect of a medication. It is important to educate nurses and patients on what are the main differences between a side effect, allergies and ADR to ensure we do not over-report by mistake. Over reporting may be recommended in grey areas when we are in doubt.

Additionally, for those patients who are not comfortable using internet tools to submit the form, they should have another option such as reporting by phone. Caregivers or family members are encouraged to report for their child or for somebody else they care for. Lastly, we need to remind our patients to report any ADR related to over-the-counter (OTC) medicines in addition to prescribed medicines. Patients with ADRs related to the use of OTC medications are recommended to consult a pharmacist or be medically confirmed by a physician if possible.

ADRs, hence having a tracking centralized tool/function at each hospital, could help to track and monitor for improvement. Improved reporting will have an impact on global health and public safety.

## Data Availability

Data sharing is not applicable to this article as no datasets were generated or analysed during the current study.

## References

[1] Canada H Mandatory reporting of serious adverse drug reactions and medical device incidents by hospitals - Overview. In:2019.

[2] Organization. WH. International Drug Monitoring: The Role of National Centres. Report of WHO Meeting. Geneva, Switzerland: World Health Organization. ;1971. 4625548

[3] Baker GR, Norton PG, Flintoft V, et al. The Canadian Adverse Events Study: the incidence of adverse events among hospital patients in Canada. CMAJ : Canadian Medical Association journal = journal de l'Association medicale canadienne. 2004;170(11):1678–1686. 10.1503/cmaj.1040498 PMC408508 15159366

[4] Hazell L, Shakir SA (2006). Under-reporting of adverse drug reactions : a systematic review. Drug Saf.

[5] Olsson S, Pal SN, Dodoo A (2015). Pharmacovigilance in resource-limited countries. Expert Rev Clin Pharmacol.

[6] Li Q, Zhang SM, Chen HT (2004). Awareness and attitudes of healthcare professionals in Wuhan, China to the reporting of adverse drug reactions. Chin Med J.

[7] Ekman E, Backstrom M (2009). Attitudes among hospital physicians to the reporting of adverse drug reactions in Sweden. Eur J Clin Pharmacol.

[8] Responses from the Canadian Society of Hospital Pharmacists to Questions Related to Mandatory Reporting of Adverse Drug Reactions and Medical Device Incidents by Provincial and Territorial Healthcare Institutions. Canadian Society of Hospital Pharmacists, Jan. 2016,.

[9] “Medication Incident Data in Canada: A Strategy for More Effective Sharing and Learning.” Medication Incident Data in Canada: A Strategy for More Effective Sharing and Learning, ISMP Canada, 30 Aug. 2017, www.ismp-canada.org/download/safetyBulletins/2017/ISMPCSB2017-07-strategy.pdf.

[10] Centers for Disease Control and Prevention (CDC). Information for Clinicians on Therapeutic Options for COVID-19 Patients. https://www.cdc.gov/coronavirus/2019-ncov/hcp/therapeutic-options.html. Accessed March 28, 2020.

[11] 5 Questions to Ask About Your Medications. 5 Questions to Ask - ISMP Canadahttps://www.ismp-canada.org/medrec/5questions.htm (accessed March 31, 2020).

