# Correction to: Amid COVID-19: the importance of developing an positive adverse drug reaction (ADR) and medical device incident (MDI) reporting culture for Global Health and public safety

**DOI:** 10.1186/s40545-020-00231-5

**Published:** 2020-06-11

**Authors:** Ali Elbeddini, Aniko Yeats, Stephanie Lee

**Affiliations:** 1 Winchester District Memorial Hospital (WDMH), 556 Louise St, Winchester, ON K0C2K0 Canada; 2 Leslie Dan Faculty of Pharmacy, University of Toronto, 144 College Street, Toronto, Ontario M5S 3M2 Canada

**Correction to: J Pharm Policy Pract (2020) 13:18**

**https://doi.org/10.1186/s40545-020-00219-1**

Following publication of the original article [1], we notified of a mistake in the article’s title.

Correct article title:

Amid COVID-19: the importance of developing a positive reporting culture of adverse drug reaction (ADR) and medical device incident (MDI) for Global Health and public safety
